# P380: Hospital hygiene- a neglected issue

**DOI:** 10.1186/2047-2994-2-S1-P380

**Published:** 2013-06-20

**Authors:** R Rao, R Mani

**Affiliations:** 1 Microbiology, Apollo Hospital, Jubilee Hills, Hyderabad, India

## Introduction

Enviromental bioburdon is a potential source of healthcare associated infections (HAI) but there has been no evidence based science to authenticate this. Cleaning practices in healthcare environments is often set in place by the environmental service workers (with minimal exposure to medical concepts) and continued. Standard guidelines of cleaning process are lacking, and as there is no scientific standard to measure the effect of the process and the bioburden, it is often not addressed by medical community. Monitoring of cleanliness is by visual audit (outdated, inadequate and scientifically obsolete) which is a purely aesthetic parameter and subjective. We put in protocols for the cleaning of patient zone and high touch surfaces for the ICU after we had an outbreak of Acinetobacter baumanni infections. We added also an indigenous (detergent) mat at the entrance of the o ICU to decrease the biobuden.

## Objectives

To implement a standard domestic hygiene protocol to prevent HAI. To make cleaning an integral part of infection control activity.

## Methods

We took up a pilot project in our 18 bedded medical intensive care unit when there was an outbreak of Acinetobacter baumanni infections. We prepared a protocol for cleaning of the ICU by defining all basic (where, how, when and who) parameters. Responsibility was delegated to the nursing staff for cleaning the bedside equipment (near patient hand touch surfaces) and the environmental service workers were accountable for the rest of the area. Infection control team gave hands on training to relevant staff. The process was monitored microbiologically (aerobic colony count) and by biochemical (ATP bioluminescence) means.

## Results

Environmental follow up surveillance showed marked decrease in the colony forming units and biobuden.

There has been no outbreaks of Acinetobacter baumanni infections after the implementation and monitoring of this protocol.Awareness and compliance of the protocol is high. A well designed cleaning protocol has been set to maintain clinical environment across hospital today.

## Conclusion

Environmental contamination is recognized as an important reservoir of the epidemic strains. High standards of domestic cleaning are essential intervention in the control of HAI. Simple hygiene is only solution in this end of antibiotic era to prevent HAI.

## Disclosure of interest

None declared

